# Construction of a Vero Cell Line Expressing Human ICAM1 for the Development of Rhinovirus Vaccines

**DOI:** 10.3390/v14102235

**Published:** 2022-10-12

**Authors:** Wouter Johannes Petrus van den Braak, Bella Monica, Diana Limpens, Dedeke Rockx-Brouwer, Matthijn de Boer, Dinja Oosterhoff

**Affiliations:** Intravacc, P.O. Box 450, 3720 AL Bilthoven, The Netherlands

**Keywords:** rhinovirus, intercellular adhesion molecule 1, ICAM1, Vero cell, manufacturing cell line

## Abstract

Human rhinoviruses (HRVs) are small non-enveloped RNA viruses that belong to the Enterovirus genus within the Picornaviridae family and are known for causing the common cold. Though symptoms are generally mild in healthy individuals, the economic burden associated with HRV infection is significant. A vaccine could prevent disease. The Vero-cell-based viral vaccine platform technology was considered for such vaccine development. Unfortunately, most HRV strains are unable to propagate on Vero cells due to a lack of the major receptor of HRV group A and B, intercellular adhesion molecule (ICAM1, also known as CD54). Therefore, stable human ICAM1 expressing Vero cell clones were generated by transfecting the ICAM1 gene in Vero cells and selecting clones that overexpressed ICAM1 on the cell surface. Cell banks were made and expression of ICAM1 was stable for at least 30 passages. The Vero_ICAM1 cells and parental Vero cells were infected with four HRV prototypes, B14, A16, B37 and A57. Replication of all four viruses was detected in Vero_ICAM1, but not in the parental Vero cells. Altogether, Vero cells expressing ICAM1 could efficiently propagate the tested HRV strains. Therefore, ICAM1-expressing cells could be a useful tool for the development and future production of polyvalent HRV vaccines or other viruses that use ICAM1 as a receptor.

## 1. Introduction

Human rhinoviruses (HRVs) are small, non-enveloped RNA viruses that belong to the Enterovirus genus within the Picornaviridae family [1]. HRVs are single-stranded RNA viruses with a positive sense genome. HRVs have an icosahedral capsid comprising 60 copies of four proteins, VP1–VP4, of which VP1–3 is surface exposed and represents the major target for an immune response [2,3]. The family of HRVs, consisting of species A, B and C, consists of more than 150 antigenically distinct serotypes, largely due to the fast adaptation and changes HRVs undergo [4].

Infections of HRVs in healthy adults cause symptoms of the common cold, including a runny nose, sore throat, coughing and sneezing [5], but can also cause more severe disease. Respiratory infections caused by HRVs have, for example, been associated with exacerbations of asthma [6,7] and chronic obstructive pulmonary disease (COPD) [8,9].

The economic burden associated with HRV infections and hospitalization is enormous. In 2000, the total of direct costs from medical care and indirect costs from absenteeism and caregiving was estimated up to USD 40 billion in the USA alone [10]. This indicates a clear need for a treatment, even though the disease itself is moderate.

In the 1980s, an antiviral therapy seemed near with the development of WIN compounds 51,711 and 52,084 by Sterling–Winthrop. Both compounds bound the HRV-B14 capsid and prevented attachment of the virus to its receptor Intercellular Adhesion Molecule-1 receptor (ICAM1, also known as CD54), but were relatively serotype specific [11]. Their successor, pleconaril (also known as Picovir), was denied FDA approval in 2002 based on safety concerns [12]. Other drugs are being investigated, including protease inhibitors [13], replication inhibitors [14] and assembly inhibitors [15], but so far, none have been approved for therapeutic use [16].

A vaccine for HRVs could alleviate serious disease in asthma and COPD cases but also, in healthy individuals, a protective vaccine could reduce the burden imposed by the common cold. Vaccines against various enteroviruses have been successfully developed in the past, most famously the Inactivated and Oral Poliovirus Vaccines (OPV), which led to the eradication of Wild Polio Virus types 2 and 3 [17,18]. More recently, a monovalent vaccine against Enterovirus-A71 (EV-A71), responsible for outbreaks of Hand, Foot and Mouth Disease, was approved by the China Food and Drug Administration [19,20]. Since introduction of the vaccine the amount of HFMD cases caused by EV-A71 has dropped markedly [21], leading to vaccine research into other HFMD-causing enteroviruses [22,23,24,25] to develop a multivalent HFMD vaccine. EV-D68, previously misclassified as HRV-87 [26], caused a major outbreak of respiratory disease associated with acute flaccid myelitis in 2014 [27]. After additional outbreaks in 2016 and 2018 [28], the virus is now a target of vaccine research by different groups [29,30,31], including our own.

The usefulness of an HRV vaccine was already realized a couple of decades ago when the first rhinovirus vaccines were developed [32,33,34,35]. During the late 1960s and early 1970s, clinical trials were performed to determine the efficacy of an inactivated rhinoviral vaccine consisting of one subtype of HRV, namely HRV-13, or an inactivated multivalent vaccine consisting of ten serotypes [35,36]. The main problem associated with these particular HRV vaccines was the lack of induction of cross-protection between the different serotypes, most likely due to the low amount of inactivated virus administered [37]. Inducing immunity to heterologous HRV serotypes is still the main challenge in the development of rhinoviral vaccines to date. Until recently, an inactivated vaccine composed of 50–100 distinct HRVs to generate protection to many different serotypes was considered impossible [38]. Novel technology, however, has made the production of such a multivalent vaccine possible, as was demonstrated in 2016. A 50-valent inactivated HRV vaccine was tested in rhesus macaques and it was demonstrated that neutralizing antibodies were raised against 80–90% of the used rhinovirus types [37]. Thus, it now seems possible to produce a multivalent inactivated HRV vaccine to protect people from most HRV infections.

However, it is important to realize that a cell line used for the production of viral vaccines needs to fulfil several criteria and needs to be approved for that application. The cells used in the study by Lee et al. were Hela-H1 cells that do not fulfil these criteria for viral vaccine production [39]. Historically, vaccines were often produced by replicating the virus in human diploid cell lines [40]. The disadvantage, however, is that these cell lines can only be cultured for a limited number of passages. To overcome this limitation, continuous cell lines have been introduced in the vaccine production field. The first continuous cell line approved for the production of vaccines was the Vero cell line, which originates from an African green monkey and was developed at Chiba University in Japan [41]. Vero cells have proven to be safe and have successfully been used for vaccine production for more than 30 years. Even now, millions of vaccine doses produced on these cells are successfully administered yearly. A major advantage of Vero cells is that these cells are sensitive to infection with many different viruses [42], meaning that Vero cells can be used for the production of a number of vaccines [43,44,45,46]. Vero cells, thus, have multiple advantages as a viral vaccine producer cell line and the Vero-cell-based viral vaccine platform technology is considered a blue-print for fast-tracking vaccine development [47].

For their entry, most species A and B HRVs rely on the already briefly mentioned ICAM1 receptor [48]. Under normal circumstances, this cell surface glycoprotein is expressed in low levels on endothelial, epithelial and immune cells, but can be upregulated as a response to inflammatory stimuli [49]. In addition to HRV species, other enteroviruses make use of the ICAM receptor family with ICAM1 being utilized by various Coxsackie C viruses [50], while ICAM5 is used by EV-D68 [51]. Previously, Tomassini et al. showed that Vero cells do not express the ICAM1 receptor, but that virus infection could be established when cells were transiently transfected with a plasmid expressing ICAM1 [52]. In this study, it was confirmed that Vero cells indeed do not express the ICAM1 receptor and it was explored whether stable expression of human ICAM1 on the surface is sufficient to create a Vero cell line, susceptible to infection with HRV.

## 2. Materials and Methods

### 2.1. Cells and Culture Conditions

Vero cells obtained from WHO (10–87) originally derived from ATCC (CCL-81) were cultured in Virus Production-Serum-Free Medium (VP-SFM, Invitrogen, Landsmeer, The Netherlands) supplemented with 2 mM glutamine (Life Technologies, Landsmeer, The Netherlands) at 37 °C in a 5% CO_2_ humidified atmosphere.

The human K562 erythroleukemic cell line and RD rhabdomyosarcoma cell line were cultured in IMDM (Invitrogen, Landsmeer, The Netherlands) supplemented with 10% and 5% fetal bovine serum (FBS, PAA), respectively, and 10 U/mL penicillin–streptomycin (Gibco, Landsmeer, The Netherlands) at 37 °C in a 5% CO_2_ humidified atmosphere.

### 2.2. Construction of an HRV-B14 Virus Research Bank on RD Cells

HRV-B14 was obtained from the ATCC with an unknown titer. RD cells were plated in a T25 flask and after 24 h 25 µL of HRV-B14 was added to the cells. HRV-B14 was propagated at 33 °C in a 5% CO_2_ humidified atmosphere. After 6 days, cells and culture medium were harvested and half of the material was used to reinfect freshly plated RD cells in a T75 flask. This was expanded until the 4th reinfection where five T175 flasks were infected. Full cytopathic effect (CPE), i.e., complete detachment of all adherent cells, was observed at day five post-infection and culture medium and cells were harvested. After three freeze–thaw cycles to release HRV-B14 still present in the RD cells, virus was centrifuged at 3000× *g* r.p.m. and the supernatant was stored at −80 °C as a working virus bank. The titer (determined with the method described below) of the HRV-B14 research bank was 7.55 log_10_ CCID_50_/mL.

### 2.3. Construction of an ICAM1 Expression Plasmid

To construct a plasmid that can be used for the transfection of mammalian cells, the human ICAM1 sequence was obtained from the RDC1030 plasmid (R&D systems, Minneapolis, MN, USA), which contains the full-length sequence from the human ICAM1 gene (Accession: NP_000192). The plasmid was transformed and amplified in JM109-competent cells (Promega, Leiden, The Netherlands) and isolated with the NucleoBond Xtra Midi EF kit (Macherey-Nagel, Dueren, Germany). The human ICAM1 sequence was isolated from this plasmid by digestion with HindIII and XbaI (New England Biolabs, Ipswich, MA, USA) and was subsequently ligated into an expression plasmid pSF.CMV.PGK-Neo/G418 (Sigma, Amsterdam, The Netherlands), which was digested with the same enzymes, to create pSF.CMV-ICAM1.PGK-Neo/G418. This plasmid contains the gene-encoding human ICAM1 under a CMV promoter and also contains a G418 gene under a PGK promoter in order to select transfected cells. Integrity of the plasmid, including ICAM1 coding sequence, was confirmed by Sanger sequencing.

### 2.4. Construction of a Stably Transfected Vero Cell Line Expressing ICAM1

Vero cells were transfected with pSF.CMV-ICAM1.PGK-Neo/G418 using FuGENE HD (Promega, Leiden, The Netherlands) following manufacturer’s instructions. After two days, expression of ICAM1 was determined with flow cytometric analysis or immunohistochemistry and transfected cells were used for further experiments or selection with G418 to construct a stable cell clone.

In order to construct a stable cell clone, transfected Vero cells were plated in ten 96-well plates through limiting dilution in the presence of 50 µg/mL G418 (Roche, Woerden, The Netherlands). The IC_50_ of G418 on Vero was determined at 3.65 µg/mL from a killing curve. For the first column a concentration of 2 × 10^3^ cells was plated per well, which was diluted sequentially 1:2 into the following columns, so that column twelve contained 1 cell/well. Cells were monitored every 3 days by microscopical inspection to screen for surviving single clones. After 3 weeks, cells from wells in which single Vero-ICAM1 clones were growing were transferred to a 48-well plate and subsequently expanded to 24 wells, 6 wells and T25 flasks. Cells were passaged at 70% confluency and part of the cells was used to determine the expression of ICAM1 on the cell surface with flow cytometric analysis. Positive Vero-ICAM1 clones were further expanded in T175 flasks and cell batches were frozen. Two clones were selected for further study, Vero_ICAM1#4C4 and Vero_ICAM1#9G4.

### 2.5. Immunohistochemistry to Determine Expression of Human ICAM1

To determine the expression of ICAM1, Vero cells were fixed by treatment with ice cold 50% MeOH/50% acetone for 20 min and after washing, cells were incubated for 1 h with an antibody directed to ICAM1 at a 1:250 dilution (Ab2213, Abcam, Amsterdam, The Netherlands). After washing, cells were incubated with rabbit anti-mouse HRP (1:1000 dilution, Abcam, Amsterdam, The Netherlands) for 1 h and subsequently washed again. As a read-out, AEC ready-to-use (Abcam, Amsterdam, The Netherlands) substrate was added for 10 min. As a negative control, non-transfected Vero cells were used.

### 2.6. Antibodies and Flow Cytometry

To determine the expression of CD155 (poliovirus receptor), CAR and ICAM1 on the parental Vero cell line and the ICAM1-expressing clones, flow cytometric analysis was performed. As a positive control for ICAM1, K562 cells were taken along. PE-labelled antibodies directed against human CD155 (eBioScience, Landsmeer, The Netherlands) or ICAM1 (BDPharmingen, Vianen, The Netherlands) were used for flow cytometric analyses, with PE-labelled IgG1 antibodies (BDPharmingen, Vianen, The Netherlands) as isotype controls. Cells were harvested from their culture vessels and suspended in PBS supplemented with 0.1% BSA and 0.02% sodium-azide. Antibody staining was performed in this buffer for 30 min at 4 °C. After washing cells, the stained cells were analyzed on a Guava EasyCyte flow cytometer (Merck Millipore, Amsterdam, The Netherlands) or an Attune NxT flow cytometer (Thermo Fisher, Landsmeer, The Netherlands). Data were further analyzed using FlowJo™ software (BD, Ashland, OR, USA).

### 2.7. Propagation of HRV-B14, HRV-A16, HRV-B37 and HRV-A57 on Vero_ICAM1

As a proof-of-principle experiment to determine whether ICAM1 expression rendered Vero cells susceptible to infection with human rhinovirus B14, Vero cells were first transiently transfected with ICAM1. Forty-eight hours after transfection these cells were infected with HRV-B14, produced on RD cells, at an MOI of 1. Virus replication was monitored for three days by scoring the percentage of CPE.

Stably transfected Vero_ICAM1 cells were used to produce an HRV-B14 batch. For this, T25 flasks with 1 × 10^6^ Vero_ICAM1#4C4 or Vero_ICAM1#9G4 cells were infected with HRV-B14. Virus propagation was performed at 33 °C in a 5% CO_2_ humidified atmosphere. After six days, cells and culture medium were harvested and after freeze thawing three times half of the material was used to reinfect freshly plated Vero_ICAM1 cells in a T75 flask. HRV-B14 was expanded until the 4th reinfection when four T175 flasks were infected. Full CPE was observed on day six at which time the culture medium and cells were harvested. After three rounds of freeze thawing followed by centrifugation, the virus was collected and stored at −80 °C as a working virus bank. The titers of the obtained virus batches were determined with the virus titration assay.

To determine whether other HRV subtypes could also replicate on Vero_ICAM1, 25 µL of HRV-A16, HRV-B37 or HRV-A57 (ATCC, titers unknown) was used to inoculate Vero cells or Vero_ICAM1#4C4 cells. As a control, Enterovirus 71 (EV71_C4) was included which replicates efficiently in Vero cells. After seven days, supernatant was harvested and used to reinfect freshly plated cells. This was repeated for three times. Pictures were taken on day six after the third reinfection.

### 2.8. Growth Kinetics of HRV-B14 on RD and Vero_ICAM1

To determine and compare the growth kinetics of HRV-B14 on both ICAM1 cell lines as well as RD cells, five T25 flasks were seeded with 1.5 × 10^6^ cells per cell line. After 24 h incubation at 37 °C all flasks were infected with HRV-B14 at an MOI of 0.01. Virus propagation was performed at 33 °C. At 1, 2, 3, 6 and 7 days post-infection one flask was harvested per cell line. Virus was released from the cells by three rounds of freeze thawing. After centrifugation, the virus samples were collected and stored at −80 °C. Once the final time point was acquired all samples were titrated using the virus titration assay.

### 2.9. Virus Titration Assay

To determine the HRV-B14 virus titer, end-point titration was used. In short, RD, Vero_ICAM1#4C4 or Vero_ICAM1#9G4 cells were seeded at a concentration of 1 × 10^4^ cells/100 µL in 96-well flat bottom plates in VP-SFM. Serial 10-fold dilutions were prepared from the various virus strains and 50 µL of each of these dilutions were added to six separate wells. After an incubation period of seven days at 33 °C cells were scored for the presence or absence of CPE and the viral titer was determined using the Reed and Muench method [53], thereby calculating the 50% cell culture infective dose (CCID_50_).

## 3. Results

### 3.1. Vero Cells Do Not Express ICAM1, the Receptor for HRV

**Figure 1:**
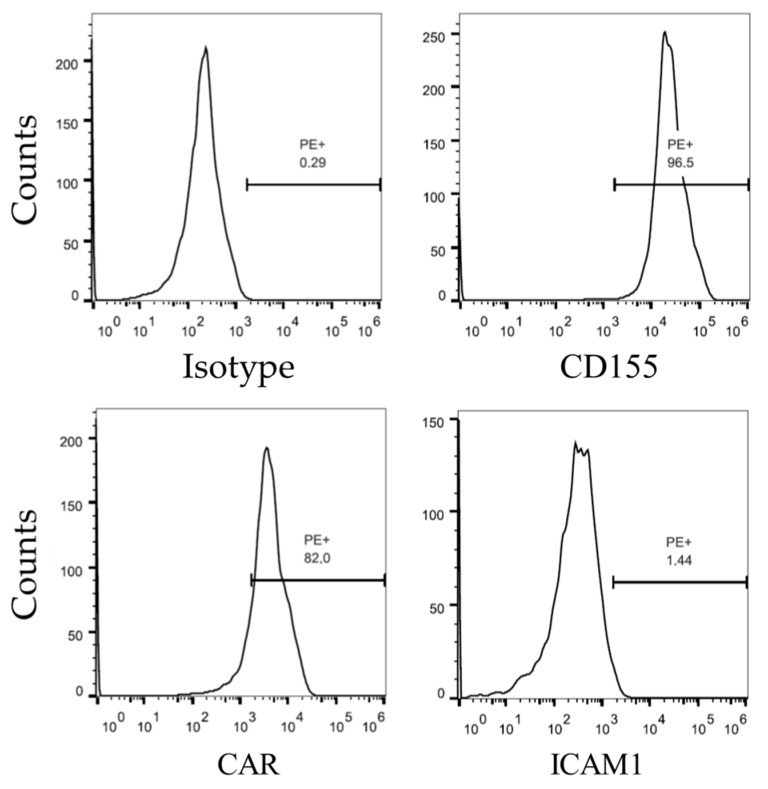
Basal surface expression of ICAM1, CD155 (the poliovirus receptor) and CAR on Vero cells as determined by staining with PE-labelled antibodies followed by flow cytometry. Vero cells express both CD155 and CAR on the surface but do not express ICAM1. This is indicated by the percentage of PE-positive cells in the right part of each graph.

To determine whether HRV can replicate in Vero cells, cells were inoculated with HRV-B14 (produced on RD cells), HRV-A16, HRV-B37 and HRV-A57 (ATCC, titers unknown). Based on microscopical inspection of CPE, no signs of viral replication were observed for any of the viruses. Surface analysis of the Vero cells by flow cytometry confirmed that the Vero cells do express CD155, the receptor for poliovirus, as well as Coxsackie and Adenoviral Receptor (CAR). However, parental Vero cells do not express detectable levels of ICAM1, the receptor for HRV (Figure 1).

### 3.2. Transient Expression of ICAM1 Renders Vero Cells Susceptible to Replication of HRV-B14

**Figure 2:**
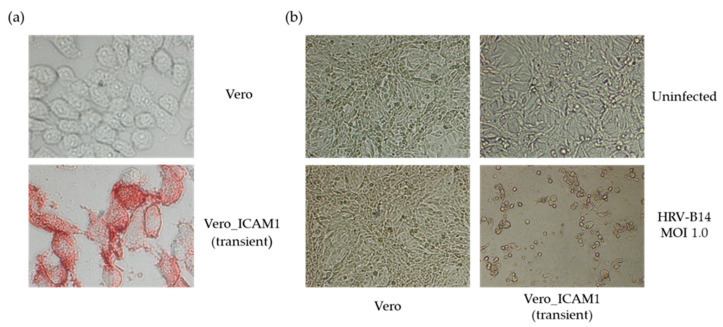
Expression of ICAM1 renders Vero cells susceptible to infection with HRV-B14. (**a**) Transient transfection of a plasmid encoding ICAM1 induces expression of ICAM1 in Vero cells as determined with immunohistochemistry; (**b**) infection of ICAM1 transiently transfected Vero cells with HRV-B14 induces replication of the virus as determined by the observation of CPE, whereas HRV-B14 cannot replicate in wild-type Vero cells. The upper row shows uninfected cells which function as negative control.

To determine whether Vero cells can support replication of HRV-B14 when ICAM1 is expressed on the surface, transient transfections using an ICAM1-encoding plasmid were performed. Vero cells transfected transiently with ICAM1 were shown to express ICAM1, as determined with immunohistochemistry (Figure 2a). Two days after transfection with the ICAM1 gene, Vero cells were infected with HRV-B14 at an MOI of 1 and viral replication was monitored for three days. Figure 2b shows that virus-induced CPE was present in ICAM1-transfected cells, but not in the parental Vero cells. These results suggest that surface expression of ICAM1 renders Vero cells susceptible to HRV-B14 infection.

### 3.3. Construction of Vero Clones Stably Expressing ICAM1

**Figure 3:**
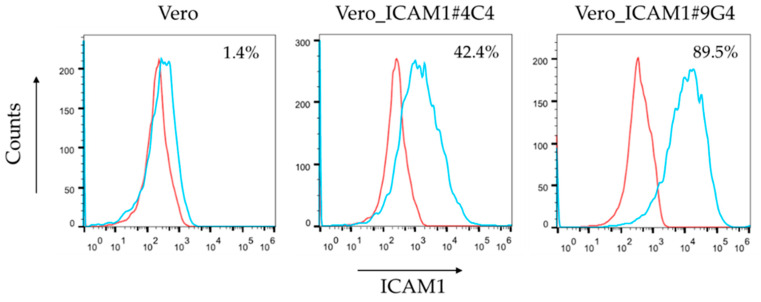
Level of ICAM1 on the surface of two stably transfected Vero clones. Expression of ICAM1 on the cell surface of two selected clones (Vero_ICAM1#4C4 and Vero_ICAM1#9G4), as determined by flow cytometric analyses. In red, staining with a PE-labelled isotype antibody and, in blue, staining with ICAM1-specific antibody. Both clones show ICAM1 expression, with highest expression in Vero_ICAM1#9G4. Percentages indicate proportion of cells expressing ICAM1.

Following limited dilutions and daily monitoring of cell growth, two clones, Vero_ICAM1#4C4 and Vero_ICAM1#9G4, which expressed high levels of ICAM1 on the cell surface, as determined by flow cytometry (Figure 3), were isolated. These clones were selected and cell banks were made. After thawing a vial from each of the cell banks, cell growth was monitored for more than 30 passages with and without G418 as a selection agent. For both Vero_ICAM1#4C4 and Vero_ICAM1#9G4, ICAM1 was still highly expressed after more than 30 passages without G418 (data not shown) and no morphological cell abnormalities were observed by microscopy (using 100× magnification). This indicates that insertion of the ICAM1 gene in the Vero cell genome is stable. Furthermore, expression of ICAM1 did not impact the growth of the cells, since the transfected cell lines showed a maximum cell density (1.7 × 10^5^ cells/cm^2^) and specific growth rate (0.022 h^−1^) comparable to the parental Vero cell line.

### 3.4. Replication of HRV on Vero Clones Stably Expressing ICAM1

**Figure 4:**
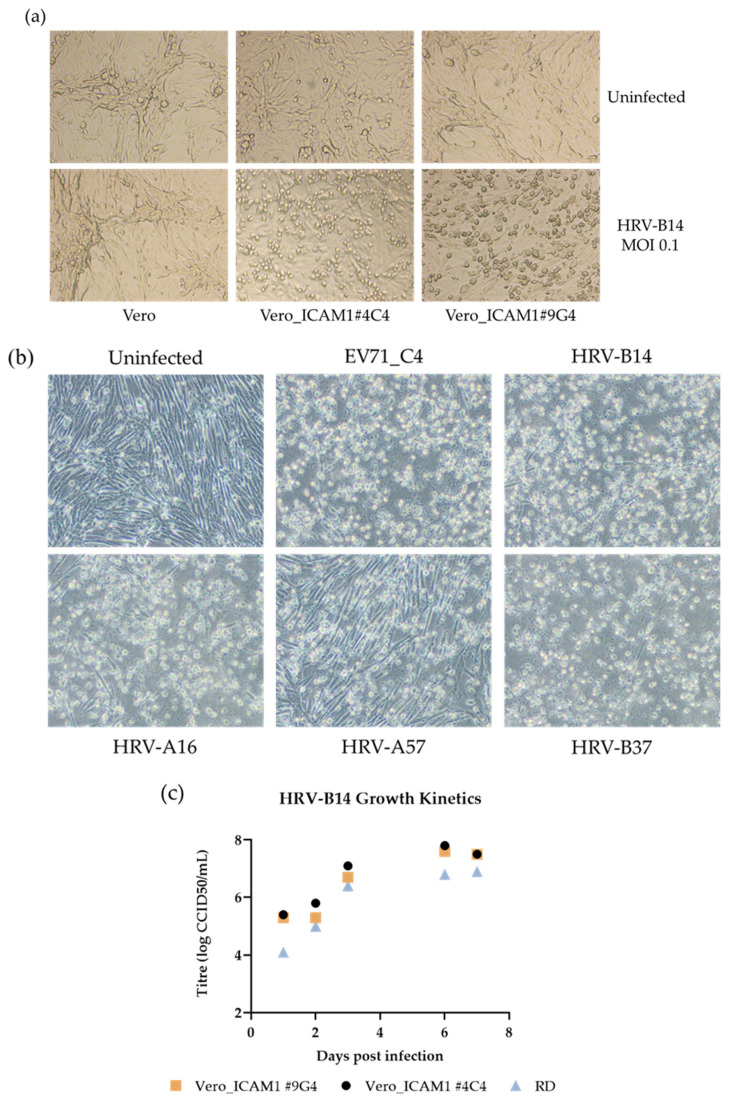
Vero_ICAM1#4C4 cells stably expressing ICAM1 can be infected with HRV-B14, HRV-A16, HRV-A57 and HRV-B37. (**a**) Infection of Vero_ICAM1#4C4 and Vero_ICAM1#9G4 with HRV-B14 at an MOI of 0.1 induces replication in the ICAM1-expressing clones, but not in the parental Vero cells, indicated by the presence of CPE in both clones; (**b**) HRV-A16, HRV-A57 and HRV-B37 can replicate in Vero_ICAM1#4C4 cells. HRV-B14 and EV71_C4 were included as positive controls; (**c**) growth kinetics of HRV-B14 infection on Vero_ICAM1#4C4, Vero_ICAM1#9G4 and RD cells after infection at an MOI of 0.01.

After successfully generating stable Vero clones, their susceptibility to infection with HRV-B14 was determined. Seven days after infection (MOI of 0.1), replication of HRV-B14 was observed in both Vero_ICAM1 cell lines (Vero_ICAM1#4C4 and Vero_ICAM1#9G4), but not in the parental Vero cell line (Figure 4a). To determine whether alternative HRV types that use ICAM1 as their entry receptor could replicate on the ICAM1-expressing Vero cells, Vero_ICAM1#4C4 was infected with HRV-A16, HRV-B37 and HRV-A57. As positive controls, EV71_C4 (in-house rescued and propagated on Vero cells) and HRV-B14 were included. After three passages, which were needed to establish productive infections, all rhinoviruses tested could replicate efficiently on Vero_ICAM1#4C4 (Figure 4b). Titers for these early passages of HRV-A16, HRV-B37 and HRV-A57 were determined at, respectively, 6.1, 6.7 and 4.9 log_10_ CCID_50_/mL on Vero_ICAM1#4C4.

In order to compare the growth kinetics of HRV-B14 on Vero_ICAM1#4C4, Vero_ICAM1#9G4 and RD cells, these cell lines were infected with HRV-B14 (MOI 0.01) and samples at different days post-infection were titrated (Figure 4c). The obtained virus titers demonstrate that HRV-B14 can replicate efficiently on Vero_ICAM1#4C4 and Vero_ICAM1#9G4, reaching titers slightly higher than on RD cells. Finally, two HRV-B14 virus batches were generated. After infection with HRV-B14 from ATCC (titer unknown) and four subsequent passages, two HRV-B14 batches were obtained with a titer of 6.8 and 6.3 log_10_ CCID_50_/mL for Vero_ICAM1#4C4 and Vero_ICAM1#9G4, respectively.

## 4. Discussion

In 2016, the interest in developing a multivalent inactivated HRV vaccine was renewed. It was then demonstrated that with modern technology, the production of a multivalent vaccine consisting of 50 HRV serotypes is feasible and vaccination of macaques induced neutralizing antibodies to all but one of the rhinovirus types present in the vaccine [37].

Crucial for developing a vaccine for human use is the availability of a cell line that results in high yields and is registered to be safe. Only a few cell lines are currently approved by regulatory agencies for the production of human vaccines. Of these, Vero cells have become the cell line of choice for many vaccine manufacturers for the production of viral vaccines, since they have proven to be safe and many different viruses can effectively replicate in these cells. However, most HRV strains, using the ICAM1 receptor, cannot replicate in Vero cells. We hypothesized that this could be overcome by constitutively expressing the receptor for HRV group A and B, ICAM1 (also known as CD54). Therefore, we generated two Vero clones that express ICAM1 at a high level on the cell surface. We showed that, indeed, this rendered the Vero cells susceptible to infection with and replication of HRV-B14. With an unoptimized process, it proved feasible to amplify the virus to high titers of up to 6.8 log_10_ CCID_50_/mL. Lee and colleagues calculated that for an 83-valent vaccine, one would require HRV stocks with titers of ≥7.0 log_10_ CCID_50_/mL [37]. Furthermore, it was shown that multiple HRV serotypes from groups A and B could replicate on the Vero_ICAM1 cell lines.

Because group C HRVs have a significant role in disease, ideally, a multivalent HRV vaccine would also include group C HRVs. Group C rhinoviruses, however, do not use ICAM1 as a primary receptor, but enter the host cell via binding to CDHR3 [54]. It remains to be determined whether Vero cells are susceptible to infection with group C HRVs and whether this is dependent on CHDR3 expression. A Vero cell line that expresses both the ICAM1 and CDHR3 receptors could be constructed to obtain an HRV vaccine producer cell line susceptible to infection with all HRV serotypes.

The ICAM1-expressing cell line could also serve as a platform for production of other viruses, since ICAM1 is also the receptor for multiple coxsackieviruses, such as CV-A15, CV-A18, CV-A21 and CVA24v [50,55,56]. Most of these coxsackieviruses do not cause disease in humans, with the exception of CVA24v, which causes conjunctivitis [57]. It is, however, well known that enteroviruses have the capacity to recombine at a high level [58] and viruses that are currently not a threat to human health could develop into more severe pathogens in the coming years. ICAM1 using viruses has also gained interest as an agent for oncolytic virotherapy. Because many tumors express ICAM1 at high levels, ICAM1 using coxsackieviruses, such as CVA21, preferentially replicate in these tumor cells, thereby inducing tumor cell lysis (reviewed in [59]). The oncolytic virus CVA21 is currently being tested in several clinical trials [60]. Therefore, the Vero_ICAM1-expressing cell line could also be considered as a producer cell line for these oncolytic viruses.

Although the parental Vero cell line is approved for the production of viral vaccines, genetic modification of the cell line for overexpressing a receptor has regulatory impact. Therefore, a new Vero cell line cannot be directly used for clinical production of viral vaccines. Most importantly, it needs to be confirmed that expression of the receptor does not have an effect on the tumorigenicity of the cell line. However, using an established and registered parental Vero cell line that is fully characterized as the starting material could possibly expedite the registration process. For example, some tests, such as viral safety, will likely not have to be repeated; this is a clear advantage over using a completely new cell line. Furthermore, using modern technology, such as CRISPR-Cas9, and the lowered costs of sequencing, it is now possible to insert transgenes at specific regions in the genomic DNA, limiting the risk of insertional oncogenesis.

Overall, a Vero cell line registered for vaccine production was successfully modified to express ICAM1 on the surface. This expression was shown to be stable for at least 30 passages. Two group A HRVs, as well as two HRVs belonging to group B, all using ICAM1 as a receptor and unable to grow on wild-type Vero cells, were successfully cultured on these modified cells. This shows that modified Vero cells could be a useful tool for the production of HRV vaccines and indicates that modified Vero cells might be of broader interest for other applications, both in the field of infectious disease as well as oncology.

## Data Availability

Data are contained within the article.

## References

[1] Taxonomy, International Committee on Taxonomy of Viruses. https://ictv.global/taxonomy.

[2] Rossmann M.G., Arnold E., Erickson J.W., Frankenberger E.A., Griffith J.P., Hecht H.J., Johnson J.E., Kamer G., Luo M., Mosser A.G. (1985). Structure of a human common cold virus and functional relationship to other picornaviruses. Nature.

[3] Sherry B., Mosser A.G., Colonno R.J., Rueckert R.R. (1986). Use of monoclonal antibodies to identify four neutralization immunogens on a common cold picornavirus, human rhinovirus 14. J. Virol..

[4] Esneau C., Bartlett N., Bochkov Y.A., Bartlett N., Wark P., Knight D. (2019). Chapter 1—Rhinovirus structure, replication, and classification. Rhinovirus Infections.

[5] Gwaltney J.M., Hendley J.O., Simon G., Jordan W.S. (1966). Rhinovirus infections in an industrial population. I. The occurrence of illness. N. Engl. J. Med..

[6] Johnston N.W., Johnston S.L., Duncan J.M., Greene J.M., Kebadze T., Keith P.K., Roy M., Waserman S., Sears M.R. (2005). The September epidemic of asthma exacerbations in children: A search for etiology. J. Allergy Clin. Immunol..

[7] Johnston S.L., Pattemore P.K., Sanderson G., Smith S., Lampe F., Josephs L., Symington P., O’Toole S., Myint S.H., Tyrrell D.A. (1995). Community study of role of viral infections in exacerbations of asthma in 9–11 year old children. BMJ.

[8] Seemungal T., Harper-Owen R., Bhowmik A., Moric I., Sanderson G., Message S., Maccallum P., Meade T.W., Jeffries D.J., Johnston S.L. (2001). Respiratory viruses, symptoms, and inflammatory markers in acute exacerbations and stable chronic obstructive pulmonary disease. Am. J. Respir. Crit. Care Med..

[9] Papi A., Bellettato C.M., Braccioni F., Romagnoli M., Casolari P., Caramori G., Fabbri L.M., Johnston S.L. (2006). Infections and airway inflammation in chronic obstructive pulmonary disease severe exacerbations. Am. J. Respir. Crit. Care Med..

[10] Fendrick A.M., Monto A.S., Nightengale B., Sarnes M. (2003). The economic burden of non-influenza-related viral respiratory tract infection in the United States. Arch. Intern. Med..

[11] Smith T.J., Kremer M.J., Luo M., Vriend G., Arnold E., Kamer G., Rossmann M.G., McKinlay M.A., Diana G.D., Otto M.J. (1986). The site of attachment in human rhinovirus 14 for antiviral agents that inhibit uncoating. Science.

[12] Senior K. (2002). FDA panel rejects common cold treatment. Lancet Infect. Dis..

[13] Hayden F.G., Turner R.B., Gwaltney J.M., Chi-Burris K., Gersten M., Hsyu P., Patick A.K., Smith G.J., Zalman L.S. (2003). Phase II, randomized, double-blind, placebo-controlled studies of ruprintrivir nasal spray 2-percent suspension for prevention and treatment of experimentally induced rhinovirus colds in healthy volunteers. Antimicrob. Agents Chemother..

[14] van der Schaar H.M., Leyssen P., Thibaut H.J., de Palma A., van der Linden L., Lanke K.H., Lacroix C., Verbeken E., Conrath K., Macleod A.M. (2013). A novel, broad-spectrum inhibitor of enterovirus replication that targets host cell factor phosphatidylinositol 4-kinase IIIbeta. Antimicrob. Agents Chemother..

[15] Mousnier A., Bell A.S., Swieboda D.P., Morales-Sanfrutos J., Perez-Dorado I., Brannigan J.A., Newman J., Ritzefeld M., Hutton J.A., Guedan A. (2018). Fragment-derived inhibitors of human N-myristoyltransferase block capsid assembly and replication of the common cold virus. Nat. Chem..

[16] McLean G., Girkin J., Solari R., Bartlett N., Wark P., Knight D. (2019). Chapter 9—Emerging therapeutic approaches. Rhinovirus Infections.

[17] WHO Two Out of Three Wild Poliovirus Strains Eradicated. https://www.who.int/news-room/feature-stories/detail/two-out-of-three-wild-poliovirus-strains-eradicated.

[18] Sabin A.B. (1985). Oral poliovirus vaccine: History of its development and use and current challenge to eliminate poliomyelitis from the world. J. Infect. Dis..

[19] Mao Q.Y., Wang Y., Bian L., Xu M., Liang Z. (2016). EV71 vaccine, a new tool to control outbreaks of hand, foot and mouth disease (HFMD). Expert Rev. Vaccines.

[20] Liu S.L., Pan H., Liu P., Amer S., Chan T.C., Zhan J., Huo X., Liu Y., Teng Z., Wang L. (2015). Comparative epidemiology and virology of fatal and nonfatal cases of hand, foot and mouth disease in mainland China from 2008 to 2014. Rev. Med. Virol..

[21] Guo W., Xu D., Cong S., Du Z., Li L., Zhang M., Feng C., Bao G., Sun H., Yang Z. (2022). Co-infection and enterovirus B: Post EV-A71 mass vaccination scenario in China. BMC Infect. Dis..

[22] Lim H., In H.J., Lee J.A., Yoo J.S., Lee S.W., Chung G.T., Choi Y.K., Chung J.K., Cho S.J., Lee J.W. (2018). The immunogenicity and protection effect of an inactivated coxsackievirus A6, A10, and A16 vaccine against hand, foot, and mouth disease. Vaccine.

[23] Sun Y.S., Xia Y., Xu F., Lu H.J., Mao Z.A., Gao M., Pan T.Y., Yao P.P., Wang Z., Zhu H.P. (2022). Development and evaluation of an inactivated coxsackievirus A16 vaccine in gerbils. Emerg. Microbes Infect..

[24] Fan S., Liao Y., Jiang G., Wang L., Zhao H., Yu L., Xu X., Li D., Zhang Y., Li Q. (2021). Efficacy of an inactivated bivalent vaccine for enterovirus 71 and coxsackievirus A16 in mice immunized intradermally. Vaccine.

[25] Zhang Z., Dong Z., Wang Q., Carr M.J., Li J., Liu T., Li D., Shi W. (2018). Characterization of an inactivated whole-virus bivalent vaccine that induces balanced protective immunity against coxsackievirus A6 and A10 in mice. Vaccine.

[26] Ishiko H., Miura R., Shimada Y., Hayashi A., Nakajima H., Yamazaki S., Takeda N. (2002). Human rhinovirus 87 identified as human enterovirus 68 by VP4-based molecular diagnosis. Intervirology.

[27] Greninger A.L., Naccache S.N., Messacar K., Clayton A., Yu G., Somasekar S., Federman S., Stryke D., Anderson C., Yagi S. (2015). A novel outbreak enterovirus D68 strain associated with acute flaccid myelitis cases in the USA (2012–2014): A retrospective cohort study. Lancet Infect. Dis..

[28] Gilrane V.L., Zhuge J., Huang W., Nolan S.M., Dhand A., Yin C., Salib C., Shakil F., Engel H., Fallon J.T. (2020). Biennial Upsurge and Molecular Epidemiology of Enterovirus D68 Infection in New York, USA, 2014 to 2018. J. Clin. Microbiol..

[29] Dai W., Zhang C., Zhang X., Xiong P., Liu Q., Gong S., Geng L., Zhou D., Huang Z. (2018). A virus-like particle vaccine confers protection against enterovirus D68 lethal challenge in mice. Vaccine.

[30] Zhang C., Zhang X., Dai W., Liu Q., Xiong P., Wang S., Geng L., Gong S., Huang Z. (2018). A Mouse Model of Enterovirus D68 Infection for Assessment of the Efficacy of Inactivated Vaccine. Viruses.

[31] Vogt M.R., Fu J., Kose N., Williamson L.E., Bombardi R., Setliff I., Georgiev I.S., Klose T., Rossmann M.G., Bochkov Y.A. (2020). Human antibodies neutralize enterovirus D68 and protect against infection and paralytic disease. Sci. Immunol..

[32] Buscho R.F., Perkins J.C., Knopf H.L., Kapikian A.Z., Chanock R.M. (1972). Further characterization of the local respiratory tract antibody response induced by intranasal instillation of inactivated rhinovirus 13 vaccine. J. Immunol..

[33] Knopf H.L., Bertran D.M., Kapikian A.Z. (1970). Demonstration and characterization of antibody in tears following intranasal vaccination with inactivated type 13 rhinovirus: A preliminary report. Investig. Ophthalmol..

[34] Knopf H.L., Perkins J.C., Bertran D.M., Kapikian A.Z., Chanock R.M. (1970). Analysis of the neutralizing activity in nasal wash and serum following intranasal vaccinaion with inactivated type 13 rhinovirus. J. Immunol..

[35] Perkins J.C., Tucker D.N., Knope H.L., Wenzel R.P., Hornick R.B., Kapikian A.Z., Chanock R.M. (1969). Evidence for protective effect of an inactivated rhinovirus vaccine administered by the nasal route. Am. J. Epidemiol..

[36] Hamory B.H., Hamparian V.V., Conant R.M., Gwaltney J.M. (1975). Human responses to two decavalent rhinovirus vaccines. J. Infect. Dis..

[37] Lee S., Nguyen M.T., Currier M.G., Jenkins J.B., Strobert E.A., Kajon A.E., Madan-Lala R., Bochkov Y.A., Gern J.E., Roy K. (2016). A polyvalent inactivated rhinovirus vaccine is broadly immunogenic in rhesus macaques. Nat. Commun..

[38] Glanville N., Johnston S.L. (2015). Challenges in developing a cross-serotype rhinovirus vaccine. Curr. Opin. Virol..

[39] Genzel Y. (2015). Designing cell lines for viral vaccine production: Where do we stand?. Biotechnol. J..

[40] Fletcher M.A., Hessel L., Plotkin S.A. (1998). Human diploid cell strains (HDCS) viral vaccines. Dev. Biol. Stand..

[41] Yasumura Y., Kawakita Y. (1963). Studies on SV40 in tissue culture-preliminary step for cancer research in vitro. Nihon Rinsho.

[42] Rhim J.S., Schell K., Creasy B., Case W. (1969). Biological characteristics and viral susceptibility of an African green monkey kidney cell line (Vero). Proc. Soc. Exp. Biol. Med..

[43] Montagnon B.J. (1989). Polio and rabies vaccines produced in continuous cell lines: A reality for Vero cell line. Dev. Biol. Stand..

[44] Montagnon B.J., Fanget B., Vincent-Falquet J.C. (1984). Industrial-scale production of inactivated poliovirus vaccine prepared by culture of Vero cells on microcarrier. Rev. Infect. Dis..

[45] Barrett P.N., Mundt W., Kistner O., Howard M.K. (2009). Vero cell platform in vaccine production: Moving towards cell culture-based viral vaccines. Expert Rev. Vaccines.

[46] Barrett P.N., Berezuk G., Fritsch S., Aichinger G., Hart M.K., El-Amin W., Kistner O., Ehrlich H.J. (2011). Efficacy, safety, and immunogenicity of a Vero-cell-culture-derived trivalent influenza vaccine: A multicentre, double-blind, randomised, placebo-controlled trial. Lancet.

[47] Barrett P.N., Terpening S.J., Snow D., Cobb R.R., Kistner O. (2017). Vero cell technology for rapid development of inactivated whole virus vaccines for emerging viral diseases. Expert Rev. Vaccines.

[48] Staunton D.E., Merluzzi V.J., Rothlein R., Barton R., Marlin S.D., Springer T.A. (1989). A cell adhesion molecule, ICAM-1, is the major surface receptor for rhinoviruses. Cell.

[49] Hubbard A.K., Rothlein R. (2000). Intercellular adhesion molecule-1 (ICAM-1) expression and cell signaling cascades. Free Radic. Biol. Med..

[50] Newcombe N.G., Andersson P., Johansson E.S., Au G.G., Lindberg A.M., Barry R.D., Shafren D.R. (2003). Cellular receptor interactions of C-cluster human group A coxsackieviruses. J. Gen. Virol..

[51] Wei W., Guo H., Chang J., Yu Y., Liu G., Zhang N., Willard S.H., Zheng S., Yu X.F. (2016). ICAM-5/Telencephalin Is a Functional Entry Receptor for Enterovirus D68. Cell Host Microbe.

[52] Tomassini J.E., Graham D., DeWitt C.M., Lineberger D.W., Rodkey J.A., Colonno R.J. (1989). cDNA cloning reveals that the major group rhinovirus receptor on HeLa cells is intercellular adhesion molecule 1. Proc. Natl. Acad. Sci. USA.

[53] Reed L.J., Muench H. (1938). A simple method of estimating fifty per cent endpoints. Am. J. Epidemiol..

[54] Bochkov Y.A., Watters K., Ashraf S., Griggs T.F., Devries M.K., Jackson D.J., Palmenberg A.C., Gern J.E. (2015). Cadherin-related family member 3, a childhood asthma susceptibility gene product, mediates rhinovirus C binding and replication. Proc. Natl. Acad. Sci. USA.

[55] Dufresne A.T., Gromeier M. (2004). A nonpolio enterovirus with respiratory tropism causes poliomyelitis in intercellular adhesion molecule 1 transgenic mice. Proc. Natl. Acad. Sci. USA.

[56] Baggen J., Hurdiss D.L., Zocher G., Mistry N., Roberts R.W., Slager J.J., Guo H., van Vliet A.L.W., Wahedi M., Benschop K. (2018). Role of enhanced receptor engagement in the evolution of a pandemic acute hemorrhagic conjunctivitis virus. Proc. Natl. Acad. Sci. USA.

[57] Mirkovic R.R., Schmidt N.J., Yin-Murphy M., Melnick J.L. (1974). Enterovirus etiology of the 1970 Singapore epidemic of acute conjunctivitis. Intervirology.

[58] Lukashev A.N. (2005). Role of recombination in evolution of enteroviruses. Rev. Med. Virol..

[59] Bradley S., Jakes A.D., Harrington K., Pandha H., Melcher A., Errington-Mais F. (2014). Applications of coxsackievirus A21 in oncology. Oncolytic Virother..

[60] Hwang J.K., Hong J., Yun C.O. (2020). Oncolytic Viruses and Immune Checkpoint Inhibitors: Preclinical Developments to Clinical Trials. Int. J. Mol. Sci..

